# Morphological Change of Heat Treated Bovine Bone: A Comparative Study

**DOI:** 10.3390/ma6010065

**Published:** 2012-12-21

**Authors:** Sumit Pramanik, Asyikin Sasha Mohd Hanif, Belinda Pingguan-Murphy, Noor Azuan Abu Osman

**Affiliations:** Department of Biomedical Engineering, Faculty of Engineering, University of Malaya, Kuala Lumpur 50603, Malaysia; E-Mails: asyikinsasha@yahoo.com (A.S.M.H.); bpingguan@um.edu.my (B.P.-M.); azuan@um.edu.my (N.A.A.O.)

**Keywords:** hydroxyapatite, scaffold, shape, crystallite, porosity, equiaxed

## Abstract

In this work, untreated bovine cortical bones (BCBs) were exposed to a range of heat treatments in order to determine at which temperature the apatite develops an optimum morphology comprising porous nano hydroxyapatite (nanoHAp) crystals. Rectangular specimens (10 mm × 10 mm × 3–5 mm) of BCB were prepared, being excised in normal to longitudinal and transverse directions. Specimens were sintered at up to 900 °C under ambient pressure in order to produce apatites by two steps sintering. The samples were characterized by thermogravimetric analysis, X-ray diffraction (XRD), and scanning electron microscopy (SEM) attached to an energy-dispersive X-ray spectroscopy detector. For the first time, morphology of the HAp particles was predicted by XRD, and it was verified by SEM. The results show that an equiaxed polycrystalline HAp particle with uniform porosity was produced at 900 °C. It indicates that a porous nanoHAp achieved by sintering at 900 °C can be an ideal candidate as an *in situ* scaffold for load-bearing tissue applications.

## 1. Introduction

Hydroxyapatite (HAp, Ca_10_(PO_4_)_6_(OH)_2_) is a main mineral constituent of bone and teeth, making up 93% of human bone with the remainder being tricalcium phosphate (Ca_3_(PO_4_)_2_, *i.e.*, β-TCP) [1,2]. The form of HAp found in bone is stoichiometric with a Ca/P ratio of 1.667. Stoichiometric porous HAp is one of the most important bioceramics for biomaterials use, owing to its high stability [3], bioactivity and biocompatibility in terms of cellular uptake [4], and it commonly has applications in the orthopaedic sectors [5,6] because it exhibits strong chemical bonding with bone [7,8]. Since a smaller size HAp crystal with highly porous structure shows very high binding ability to various biomolecules [7,8,9,10], it is being used in a wide range of important biomedical applications [6,9,11,12,13]. Thus, attempts at producing synthetic HAp have been undertaken in many ways, such as the solid state reaction [5] and wet chemistry [13,14,15,16], using various reagents [12,14], while natural HAp can be produced from corals [17], shells [18,19] and bones [1,2] for tissue engineering scaffolds as well as in many advanced applications [9,11,12,13]. However, no previous study has been undertaken to evaluate a suitable heat treatment method, which can provide a uniform porosity along with the quality and quantity of HAp for bone scaffold. Over recent decades, there have been several attempts taken to improve the osteogenic potential of xenogeneic bone for clinical use [20,21,22]. However, most of these efforts have been unsuccessful owing to undesirable biological reactions such as infection, disease transfer and immunological defensive reaction [1]. The biological reactions produced by bioceramics depend, to a significant extent, on its chemical composition, phase purity, and morphology (e.g., particle size, shape and porosity) [23]. Thus, phase modulation of HAp has been attempted through various techniques [3,5,24]. In this study, the bovine bone, readily available in nature, has been selected as a source of HAp because of its quality (purity > 95%) [1,2], quantity (>60 wt%) [2] and cost effectiveness [1]. Although HAp has been produced from bovine bone, a thorough investigation of desired morphology such as nano-porous structure [25], effective as a scaffold for load-bearing tissue engineering (TE), has not been explored to determine the appropriate temperature [2]. Additionally, no attempt has been made to predict the shape morphology of pure HAp by X-ray diffraction (XRD) study so far. Furthermore, no previous study has been able to show the complete mechanism of phase and morphological change in bovine HAp with variation in sintering temperature. The aim of the present study is to determine a proper heat treatment at which an apatite of the desired morphology for bone TE scaffold can be developed very easily. Thus, in this novel study, we report a complete analysis of change in phase and morphology of bovine-HAp under heat treatment with two step sintering. In addition, for the first time, we predict the shape morphology of HAp particles by XRD technique and eventually verify with scanning electron microscopy (SEM) analysis.

## 2. Materials and Methods

In this study, adult femoral bovine bones were purchased from the local market. The distal (near knee) part of the raw femoral bones was freed from muscles, ligaments, and cartilage macroscopically by being boiled in distilled water for 2.5 h. The boiled bones were placed in an ultrasonicator (model: SW12H, make: M/s Sono Swiss) filled with acetone (AR grade, supplied by M/s Fischer Scientific (M)) for 5 min to clean the remained fats. Rectangular shaped specimens (size: 10 mm × 10 mm × thickness; the thickness varies from 3 to 5 mm) of bovine cortical bone (BCB) were prepared, being excised normal to longitudinal and transverse directions. The each BCB sample was first, dried at 120 °C for 12 h in the oven (model: Memmet, make: M/s Naluri Scientific) and then, sintered at different temperatures such as 350, 500, 750 and 900 °C for 3 h under ambient condition using box furnace (model: L8-1200, make: M/s VISTEC Technology). For drying, the intermediate holding temperature was 70 °C for 3 h and the final drying temperature was at 120 °C for 9 h. Each heat treatment was performed in two steps, first, isothermal heating at an intermediate temperature and last, soaking at a final sintering temperature, to remove the unwanted residual phases completely. For the sintering temperatures 350, 500, 750 and 900 °C, the intermediate temperatures were held at 200, 350, 500 and 550 °C, respectively for 1 h. The heating rate up to intermediate temperature was 5 °C/min, and after intermediate to final temperature it was 10 °C/min for each sample. Then, each sample was furnace cooled to room temperature.

## 3. Experimental Section

The weight change of the *as-received* cortical part of the femoral bone was verified by thermogravimetric analysis (TGA) using thermogravimetric analyzer (model: TGA/SDTA851^e^ Ultra microbalance, make: M/s Mettler Toledo) at a constant heating rate of 10 °C/min in controlled air atmosphere.

Phase analysis, crystallite size, and residual strain in crystals of the above samples were determined by wide angle X-ray diffraction with CuK*α* radiation of wave length λ = 1.54056 Å using X-ray diffractometer (model: Empyrean, make: M/s PANalytical BV). The crystal dimension or crystallite size (*t*) and crystal strain or elastic residual strain (*η*) of the samples were calculated following modified Debye Scherrer expression Equation (1) [12,26]:

(1) $$\Delta {\text{θ}}_{\text{FWHM}}\;\text{cos}({\text{θ}}_{B})=\frac{\kappa \text{λ}}{\text{t}}+4\;\eta \;\text{sin}({\text{θ}}_{B})$$

where, *κ* be the constant (e.g., 0.9) depends on the particle morphology, *θ_B_* (in degree) be the Bragg’s angle, and Δθ_FWHM_ (in rad) be the full width at half maxima (FWHM) at 2*θ_B_*. The instrumental broadening was considered by using standard XRD of silicon wafer sample.

Morphology and crystal formation of the above heat treated samples were performed under dual beam focused ion beam (FIB)—field emission scanning electron microscope (FESEM) attached with energy dispersive analysis of X-ray (EDAX) (model: AURIGA, make: M/s Carl ZEISS) using secondary electron. The average value of at least five selected areas in EDAX was employed for each sample. All the samples for SEM and EDAX were explored as it, without any further coating, on aluminium stubs.

Bulk density (ρ, g/cc) and open porosity (P_Open_, %) were measured by the Archimedes’ principle following the Equations (2) and (3):

(2) $$\text{ρ}=\frac{{\text{m}}_{1}}{{\text{m}}_{3}-{\text{m}}_{2}}\times {\text{ρ}}_{\text{water}}^{25{}^{\circ}\text{C}}$$

(3) $${\text{P}}_{\text{Open}}=\frac{{\text{m}}_{3}-{\text{m}}_{1}}{{\text{m}}_{3}-{\text{m}}_{2}}\times 100$$

where, *m_1_* is the mass of the specimen in air, *m_2_* is mass of the specimen into normal distilled water, and *m_3_* is the mass of the wet specimen after taking out from the water. At least five identical specimens were carried out at 25 °C, where water density ( ${\text{ρ}}_{\text{water}}^{25{}^{\circ}\text{C}}$ ) was considered as 0.99704 g/mL, to evaluate the average as well as standard deviation for each sintered samples.

## 4. Results and Discussion

### 4.1. TGA

**Figure 1:**
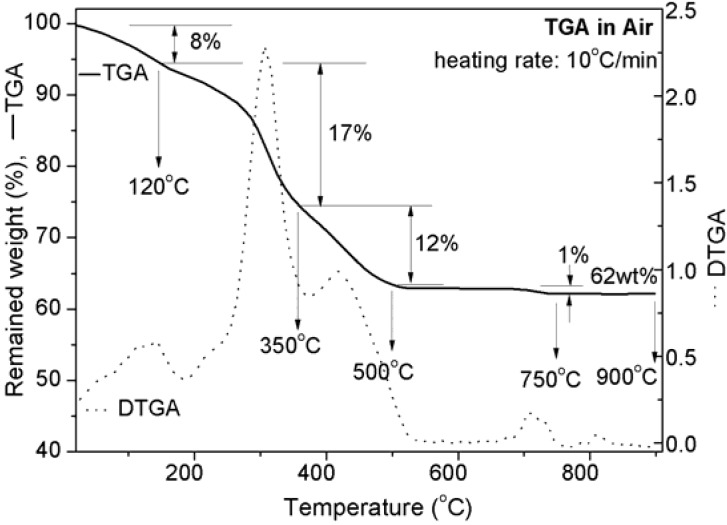
Thermal stability of the samples by thermogravimetric analysis (TGA) and derivative of thermogravimetric analysis (DTGA) in air atmosphere.

The TGA inflations as well as derivative of thermogravimetric analysis (DTGA) peaks, as depicted in Figure 1, clearly indicate the distinct weight losses of the BCB after certain temperatures. A first significant amount of weight loss (~8%) is observed up to 195 °C owing to the removing of adsorbed water and moistures. The next rapid as well as maximum weight loss (~17%) is found in temperatures up to 375 °C owing to the evaporation of a part of the organic components, including collagen polymer fibrils of bone. The third major weight loss (~12%) is noticed in temperatures up to 550 °C owing to almost complete evaporation of remained part of the organic components of bone. These total water content (8 wt%) and total organic content (29 wt%) in BCB are very much similar to the results reported in elsewhere [2]. Then a small amount of weight loss (~1%) is observed up to 775 °C owing to transformation of HAp from other phosphates. However, with further increasing of temperature, a negligible amount of weight loss is found. This indicates the complete formation of HAp crystals at up to 890 °C. Therefore, heat treatment temperatures can be fixed from the above inflations of the TGA curve or as a derivative of TGA (DTGA) curve as considered just below (e.g., 120, 350, 500 and 750 °C) the end (*i.e.*, 195, 370, 550 and 775 °C, respectively) of a particular degradation or decomposition reaction. The last sintering temperature, 900 °C, was selected just above the end of reaction (*i.e.*, 890 °C) to get a single phase of pure HAp. The TGA plot also determines the remained weight percentage, ~62%, of the cortical bovine bone at 900 °C. This implies that the maximum amount of pure HAp that can be produced by sintering in air atmosphere is >62 wt% of *as-received* cortical bovine bone, which is close to the result reported in elsewhere [2]. The little difference in total weight loss from the reported results indicates that the total HAp content in BCB may vary slightly with different aged and/or healthy bones.

### 4.2. XRD

**Figure 2:**
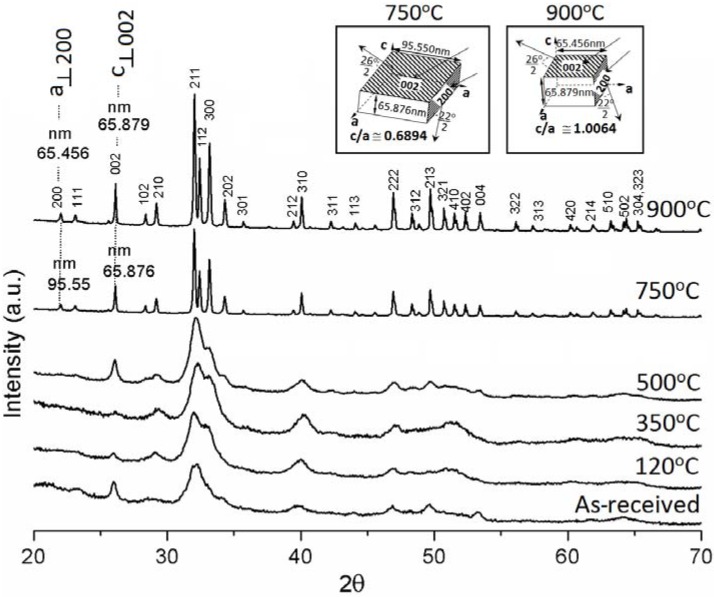
X-ray diffraction (XRD) patterns of *as-received* bovine bone and heat treated bone at 120, 350, 500, 750 and 900 °C with crystallite dimensions (insets).

**Figure 3:**
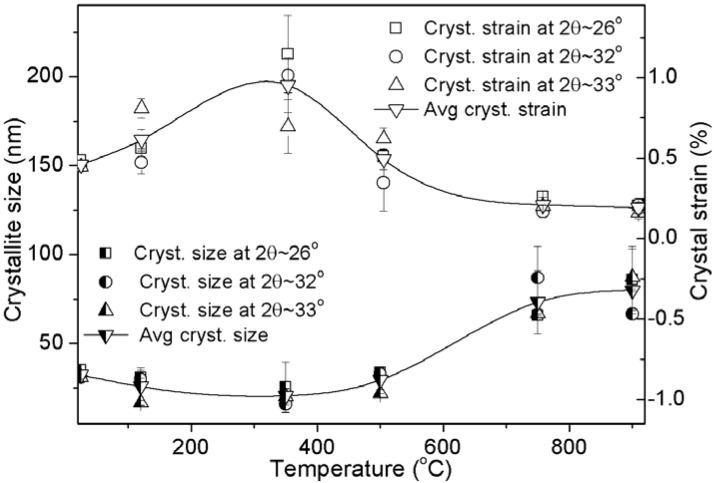
Crystallite size and crystal strain at different heat treated bovine bones including *as-received* bone.

**Figure 4:**
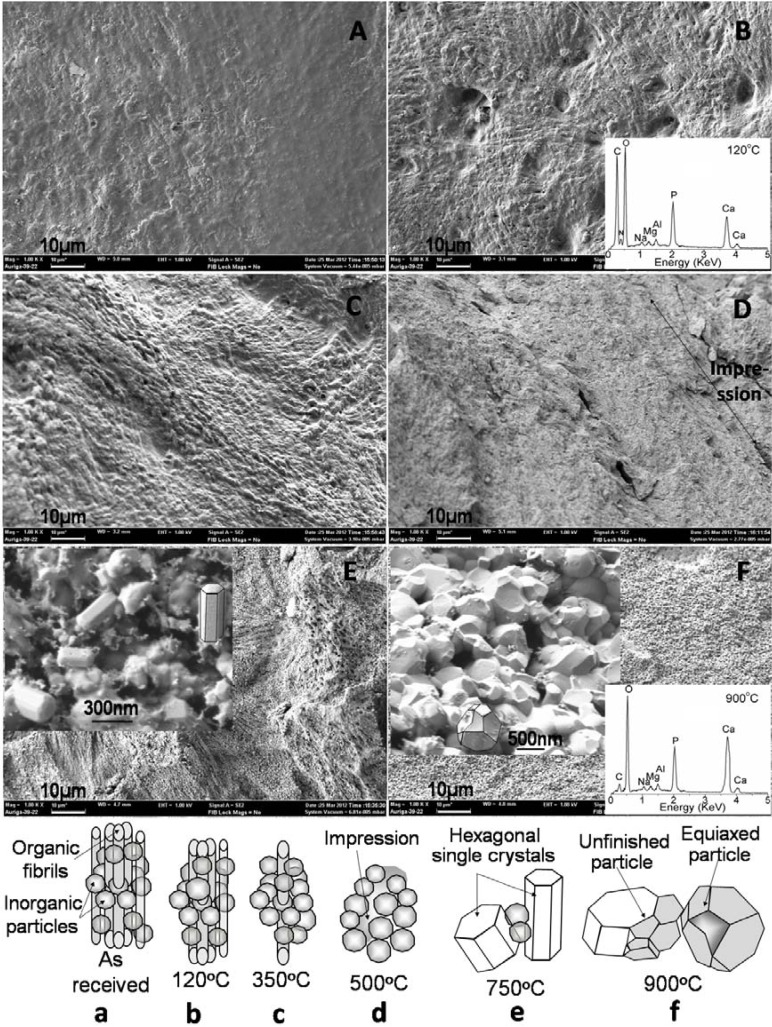
Scanning electron microscopy (SEM) micrographs and corresponding schematic steps of (**A** & **a**) *as-received* bovine bone and heat treated bone at (**B** & **b**) 120, (**C** & **c**) 350, (**D** & **d**) 500, (**E** & **e**) 750 and (**F** & **f**) 900 °C.

XRD patterns of all the heat treated BCBs including *as-received* bone are illustrated in Figure 2. The *as-received* specimen shows a broad peak with highest intensity at *2θ* ~32°. This amorphous nature is occurred because of the organic polymer part of bone that strongly interferes to the diffraction of crystalline apatite. It becomes gradually prominent by increasing of sintering temperature. This confirms that the total solid content of bovine bone is composed of apatite minerals as well as organic polymers (see the XRD patterns from *as-received* to 500 °C in Figure 2). The heat treated samples at 120 to 500 °C show the broad peak for the (211) plane with a shoulder peak for the (300) plane at *2θ* ~32° and 33°, respectively. However, after 500 °C, this broad peak gradually becomes split into to three distinct crystalline peaks, (211), (112) and (300) at *2θ* ~32°, 32.4° and 33°, respectively, which are similar to the standard HAp having hexagonal crystal system and 6_3/m_ space group [JCPDS 09-0432]. This result strongly declares the formation of crystalline pure HAp. The intensity count of XRD peaks is increased owing to the detection of more number of diffracted rays developing from the larger number of same group of planes. It is only possible when the number of crystals as well as crystalline domain planes is increased. As a result, the more number of crystalline pure HAp phase formations is revealed at 900 °C compare to 750 °C temperature as the peaks become more intense at 900 °C. This result also strongly supports our SEM result, where HAp crystal formation is found to start growing as hexagonal particle at 750 °C and transforms into equiaxed at 900 °C, will be discussed later. Crystal dimensions or crystallite sizes are evaluated by using of Equation (1). No significant difference in average crystallite size (e.g., 32.4, 25.8 and 20.5 nm for *as-received*, 120 °C and 350 °C samples, respectively) is observed up to 500 °C; and after 750 °C, it almost becomes steady to 79.9 nm at 900 °C (Figure 3). The average crystallite size increases significantly from 29.5 to 73.1 nm at 500 to 750 °C, respectively owing to maximum transformation as HAp phase from other calcium phosphate or apatite phases. Therefore, this result indicates that the major phase transformation and morphology conversion of the apatites are occurred in the range of 500 to 750 °C and 750 to 900 °C, respectively. Consequently, the average crystal strain, owing to the effect of heat treatment as well as defects present in materials, increases significantly (e.g., 0.46, 0.61, 0.95 and 0.49 for *as-received*, 120, 350 and 500 °C samples, respectively) with increasing of sintering temperature up to 500°C and then, shows a major drop to 0.21 at 750 °C followed by a steady change (*i.e.*, 0.19) at 900 °C (Figure 3). The maximum crystal strain, 0.95, is observed at 350°C owing to removing of large amount of organic polymer from the mineral phases as confirmed by the maximum weight loss (17%) from 120 to 350 °C in TGA (Figure 1). This is first time we report that the shape of crystallites can also be predicted by XRD analysis. In order to predict the crystallite shape, we have assumed that the crystal dimension at a particular plane is perpendicular to a specific crystal axis. Here, we have selected the crystal dimensions at two different specified planes, where one is perpendicular (_┴_) to the *a*-axis (*i.e.*, {200} at *2θ* ~ 22°) and another is perpendicular to the *c*-axis (*i.e.*, {002} at *2θ* ~26°), to predict the shape morphology of HAp crystals by XRD analysis (see insets of Figure 2). Two heat treated samples (*i.e.*, sintered at 750 and 900 °C temperatures) are only considered to predict the shape of crystallites since no other material has prominently shown the {200} peak. It has been found that the ratio of crystal dimensions *i.e.*, *c*_┴(002)_ to *a*_┴(200)_ ratio at 750 °C, 0.6894, is very close to the standard unit cell of HAp (*i.e.*, *c*/*a* = 0.7309 [JCPDS 09-0432]). However, the *c*_┴(002)_/*a*_┴(200)_ ratio at 900 °C, 1.0064, is nearly 1. This indicates that the high aspect ratio of hexagonal HAp crystals become equiaxed when temperature increases from 750 to 900 °C. This result strongly supports our SEM result where hexagonal particles of 750 °C are found to be converted into equiaxed polycrystalline at 900 °C (Figure 4).

### 4.3. SEM

The change in morphology of bovine bone with sintering temperatures is depicted in SEM micrographs Figure 4A**–**F, and the corresponding schematics are predicted in Figure 4a**–**f to understand the proper mechanism or step for formation of HAp with sintering temperatures. The *as-received* bone reveals densely compacted organic molecules including collagen fibrils along with strongly bonded inorganic mineral structure (Figure 4A and a). After drying at 120 °C, the fluid content including water as well as a part of the organic molecules comes out and the collagen fibrils are revealed more clearly as seen in Figure 4B and schematically represented in Figure 4b. Then after sintering at 350 °C, most of the collagen polymer molecules get melt and very few fibrils still remain in the material; this is presented in Figure 4C and a schematic view is depicted in Figure 4c. At 500 °C, almost all the polymer fibrils are burnt out and a few large size fibrils leave impressions in the samples after complete burning out: it is shown in the SEM Figure 4D and depicted schematically in Figure 4d. Since most of the organic polymers are removed at 500 °C, the smaller size (*i.e.*, <30 nm) inorganic calcium phosphates crystals are revealed in the microstructure. This result confirms that the bovine bone is a natural composite, which comprises polymer fibrils and nanoceramic (*i.e.*, apatite). The hexagonal HAp particles, including single crystals, with wide range of dimensions (*i.e.*, 100–400 nm in length and 80–200 nm in width) are nucleated at just below at 750 °C; and it is clearly depicted in Figure 4E and e. At 900 °C, mostly, all the hexagonal crystals and particles are converted into equiaxed polycrystalline particle of size 300–550 nm (average ~400nm) having uniform interconnected porosity with pore size 0.3–1.0 µm (average ~0.5 µm) (Figure 4F and f). Hence, this result confirms that the major phase transformation occurred in between 500 and 750 °C is owing to lattice diffusion and morphology conversion in range of 750 to 900 °C is revealed via surface diffusion. The SEM study also strongly supports our XRD result as mentioned before. The uniform porosity with submicron range is ideally needed for load-bearing tissue engineering scaffolds [25].

### 4.4. EDAX

The vital elements, such as calcium (Ca), phosphorous (P), carbon (C), and oxygen (O), present in the bovine bone are identified by EDAX study. Another element nitrogen (N), 11–12 At%, is found in dried (120 °C) specimen (see the inset in Figure 4B) may be owing to the amine (-NH_2_) group of protein or peptide molecules in the organic part of the bone [27,28]. The N-atom is found to be disappeared after sintering (e.g., 900 °C) and shown as inset at bottom-right corner of Figure 4F. While C-atom of dried bone (~65 At%) is significantly reduced but still remained in 900 °C sintered HAp as around 5.5 At% as a carbonate. The other trace (≤5 At%) elements such as sodium (Na), magnesium (Mg) and aluminium (Al) are also almost constantly present in all sample as detected by the EDAX study. However, one another important element, hydrogen (H), which must be present in each specimen, couldn’t be recognized by this analysis owing to the limited detectability of the analyzer for the lower atomic number elements. The Ca/P molar ratio of dried bone, nearly 1.0 as detected by our EDAX study, is found to reach 1.62 after sintering at 900 °C, which is close to stoichiometric HAp (1.667). This result implies the formation of pure HAp phase at 900 °C.

### 4.5. Density and Porosity

Density and open porosity results also strongly support our SEM results. The bulk density of materials is quite higher for the untreated or *as-received* (~1.984 g/cc) as well as 120 °C dried (~2.0160 g/cc) bones compare to sintered bones. After sintering, it decreases to ~1.502 and 1.201 g/cc at 350 °C and 500 °C, respectively owing to removing of organic polymers which were acting as binding agent in the bone as a composite. Conversely, after sintering, these natural organic binders create porosity of the material as confirmed by the open porosity result (see Table 1). However, at 750 °C, a densification is observed up to 1.501 g/cc owing to the combination of larger apatite particles as well as newly formed HAp particles via lattice diffusion [29]. Then at 900 °C, the density (1.353 g/cc) is slightly decreased via surface diffusion, and it provides a more uniform porous microstructure that was shown in the SEM image (Figure 4F). With an increase in temperature, the density may increase, but porosity would decrease significantly owing to grain growth. Thus, the *in situ* scaffold of HAp sintered at 900 °C having uniform porosity can be potential candidate for tissue engineering [25].

**Table 1:** Bulk density and open porosity of as-received and heat treated bones.

Heat treatment of sample	Bulk Density ± SD (g/cc)	Open porosity ± SD (%)
As received	1.984 ± 0.009	2.278 ± 0.766
120 °C	2.016 ± 0.053	2.309 ± 0.046
350 °C	1.507 ± 0.060	15.724 ± 2.918
500 °C	1.201 ± 0.138	33.023 ± 0.453
750 °C	1.501 ± 0.002	2.039 ± 0.878
900 °C	1.353 ± 0.029	10.275 ± 1.451

Note: SD: Standard deviation

## 5. Conclusions

In order to prepare a desired morphology of bovine-HAp for load-bearing scaffolds, only cortical part of the femoral bovine bone was studied. This study has determined easily a proper heat treatment temperature from the inflations of TGA curve and DTGA peak to get a proper morphology of single phase HAp. For the first time, we have predicted the morphology or shape of crystallites by XRD technique. The XRD result has shown that a major phase transformation as well as morphology conversion of bone occurs in the ranges of 500 to 750 °C and 750 to 900 °C, respectively. The mechanism or step for formation of the HAp crystals from heat treated BCB has been revealed by SEM micrographs as well as density test. The equiaxed and pure polycrystalline HAp with uniform porous microstructure can be produced from heat treated bovine bone at 900 °C. The morphology, including shape and porosity, which is the most important factor for the TE scaffold, has also been optimised by heat treatment. Recently, we have found that the crystalline materials show improved mechanical properties [28,30] and the interconnected porous structure shows better bioactivity [25]. Therefore, we predict that the crystalline pure HAp having uniform interconnected porosity of submicron range, which was successfully developed from heat treated bovine bone sintered at 900 °C, would be a potential candidate for load-bearing tissue engineering scaffolds.
